# Targeted CYP2E1 quantification and its correlation to currently acceptable clinical biochemical indices

**DOI:** 10.1186/s40709-016-0052-9

**Published:** 2016-06-30

**Authors:** Christina Gertrude Yap, Anuar Zaini, Iekhsan Othman

**Affiliations:** Jeffrey Cheah School of Medicine and Health Sciences, Monash University Malaysia, Pesiaran Lagoon Selatan, 46150 Petaling Jaya, Selangor Malaysia

**Keywords:** Absolute quantitation, CYP2E1, Diabetes progression, Early risks predictor, LCMS/MS/MS, ROS

## Abstract

**Background:**

The Cytochrome P450 enzymes are commonly known for their major role in metabolism. Besides its metabolic role, CYP2E1 gene expression has been associated with the onset of diabetic nephropathy. CYP2E1 protein elevation has also been reported to be responsible for the production of reactive oxygen species. The aims of this study were (i) to optimize and validate a targeted proteomic approach for quantitating CYP2E1 and validating it as a suitable clinical test, (ii) to investigate the concurrency between ESI-LCMS-MS quantitated circulating CYP2E1 and gold standard indices in the context of outpatient point-of-care clinical settings involving various groups of diabetic patients and (iii) to investigate the concurrency profile of circulating CYP2E1 protein, CYP2E1 gene expression and reactive oxygen species (ROS). This is a cross sectional study involving three groups of subjects (n = 166): control, pre-diabetes, and diabetes. We optimized a targeted proteomic approach for absolute quantification of CYP2E1. “YPEIEEK” and “GTVVVPTLYDNQEFPDPEK” were the representative peptides of CYP2E1 for our analytical method. Deuterated forms of “YPEIEEK” and “GTVVVPTLYDNQEFPDPEK” were used as internal standards. Lymphocytes were isolated from whole blood, microsomes were prepared, followed by in-solution digestion for production of tryptic peptides. Amounts of “YPEIEEK” and “GTVVVPTLYDNQEFPDPEK” from patients’ samples were calculated from a calibration curve.

**Results:**

“YPEIEEK” is a unique and reliable representative peptide for CYP2E1 quantification. “GTVVVPTLYDNQEFPDPEK” showed poor reproducibility and sensitivity. Incremental amounts of CYP2E1 protein in the peripheral circulation clearly showed concurrency with CYP2E1 gene expression and ROS levels in our study population. Elevations of CYP2E1 were observed even when gold standard clinical indicator for glycemic control (HbA1c) was within normal reference limits. Quantitated amounts of CYP2E1 protein in the pre-diabetes and diabetes groups showed significant difference relative to control group *(p* < 0.001). No significant differences were observed between the medians of pre-diabetes and diabetes groups (*p* = 0.870).

**Conclusions:**

CYP2E1 protein in peripheral blood can be reliably quantitated by the validated targeted proteomic approach method. Quantifiable amounts of CYP2E1 preceded abnormal HbA1C levels which indicates quantitation of CYP2E1 could be useful as an additional tool for early indication of diabetic risks and it complications.

## Background

Cytochrome P450 (CYP450) is a superfamily of metabolic enzymes commonly known for their primary role in the metabolism of endobiotics and xenobiotics [1]. The main normal tissue in which CYP450s are expressed is in the hepatocytes [2]. Specific forms of CYP450 are present in extra-hepatic tissues such as the intestines, lungs and lymphocytes [2]. However, peculiar extra-hepatic expressions of specific individual CYP450 isoforms have been associated with various pathological conditions such as in organ specific cancers [3, 4] and tumors [5], diabetes [6–8] and chronic kidney diseases [9]. The CYP1B1 subtype is tumour specific, while the CYP1A family has been identified predominantly in several types of organ cancers and CYP3A5 is the predominant subtype identified in lung tumour as well as in lung cancer cells [2]. Recent studies have also showed that CYP2E1 expression in peripheral lymphocytes [7, 8] is elevated in diabetes and kidney disorders [9]. The correlation between CYP2E1 level in peripheral blood lymphocytes and early pathogenesis of diabetic nephropathy has recently been demonstrated [10]. Notably, besides the important role that CYP450 superfamily plays in xenobiotics and endobiotics metabolism, individual CYP450 isoforms may pose as candidate markers of a specific disease or pathology [11]. If the proposed candidate markers are measurable earlier than conventional clinical indices, the former may be useful as early indicators of an onset of particular disease. Early indicators of diseases are essential for prevention of further progression and for early planning of effective interventions.

Rapid methods to quantitate these diseases’ specific CYP450 isoforms will allow early diagnosis, prognosis or monitoring a particular disease or may be used alongside conventional clinical tests as an alternative test for confirmation. Many methods have been described for qualitative and quantitative identification of CYP2E1. Earlier common qualitative methods for detection and identification of CYP450 subtypes were gel electrophoresis [12] and immunoblotting [13, 14] which lacked sensitivity and specificity. In the past decade, the quantitative proteomic approach gained popularity and many studies have attempted the application of trypic digest coupling with tandem LCMS analysis [15, 16] allowing absolute quantitation of individual CYP450 isoforms. These approaches demonstrated higher specificity and sensitivity but to date (and according to our knowledge) no studies have described a validated targeted proteomic method for analyzing CYP450s in human peripheral blood. Since a few isoforms of CYP450s have been associated with diseases, it will be worthwhile introducing a clinically applicable method for quantitating CYP450s.

Although various studies have convincingly dictated the association of CYP2E1 [6–10] and reactive oxygen species (ROS) [17] in diabetes and its complications, there is lack of evidence on the changing profile of ROS and CYP2E1 levels with varying glycemic levels. Also, there is lack of evidence on the concurrency of ROS and CYP2E1 with gold standard clinical indices for glycemic control (HbA1c). The knowledge of the latter will further reaffirm that CYP2E1 is a candidate early biomolecule in diabetes progression. Therefore, the aims of this study were firstly, to optimize a targeted proteomic approach for quantitating CYP2E1 in peripheral blood; secondly, to validate its use alongside with clinical indices (HbA1c and fasting blood sugar) currently used in the clinical laboratory, and thirdly, to investigate the correlation of circulating CYP2E1 protein amounts with CYP2E1 gene expression and ROS.

## Results and discussion

**Fig. 1:**
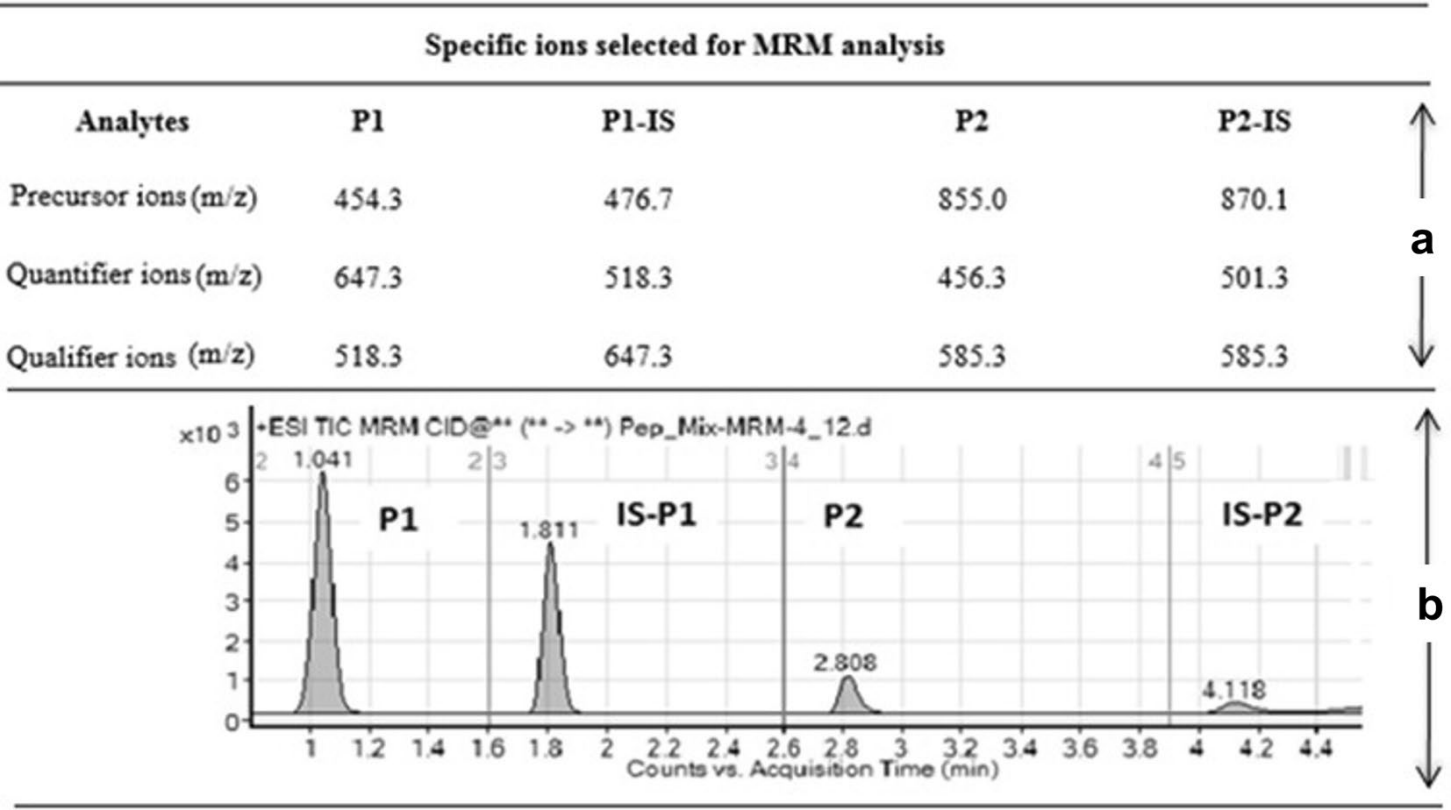
Representative chromatograms of analytes: YPEIEEK (P1), GTVVVPTLDSVLYDNQEFPDPEK (P2) and internal standards for YPEIEEK (P1-IS) and GTVVVPTLDSVLYDNQEFPDPEK (P2-IS). **a** 30 fg µL^−1^ mixture of P1, P2, P1-IS and P2-IS were prepared in pooled plasma from control subjects and injected into the Agilent 1100 UHPLC coupled to the Agilent 6410 ESI-LCMS/MS system for CYP2E1 analytical method development and optimization. Stable and reproducible transition ions (*m/z*) were selected for MRM analysis: P1 = (precursor: 454.3; quantifier: 647.3; qualifier: 518.3); P1-IS = (precursor: 476.7; quantifier: 518.3; qualifier: 647.3); P2 = (precursor: 855.0; quantifier: 456.3; qualifier: 585.3); P2-IS = (precursor: 807.1; quantifier: 501.3; qualifier: 585.3). *MRM* multiple reaction monitoring; *P1* peptide 1 (YPEIEEK); *P1-IS* internal standard for peptide 1; *P2* peptide 2 (GTVVVPTLDSVLYDNQEFPDPEK); *P2-IS* internal standard for peptide 2. **b** Representative chromatogram showing good resolution of each analyte of interest from a mixture containing 30 fg µL^−1^ of each analyte spiked pooled plasma from control subjects. The specific retention time for each analyte was P1: 1.041 min; IS-P1: 1.811 min; P2: 2.808 min and P2-IS: 4.118 min. Total run time for each sample was 5 min

The representative peptides for CYP2E1 used in this study were YPEIEEK [P1 (amino acid position 318–325)] and GTVVVPTLYDNQEFPDPEK [P2 (amino acid position: 386-408)]. P1 is suitable for universal quantification of CYP2E1 in human due to absence of polymorphic regions. P2 was included as a confirmatory peptide to the former because it has been employed in other studies [15]. P2 may not be suitable as a representative peptide for universal application in CYP2E1 quantification due to natural amino acid variation between individuals at position 389 [URL: http://www.uniprot.org]. An electrospray ionization source (ESI) was operated in the positive ionization mode for ion production. The most abundant and stable product ions from collision-induced dissociation of each analyte were selected as quantifier ions, while the second most abundant ions were selected as qualifier ions (Fig. 1a). An excellent separation of each analyte of interest was achieved with total run time for each injection of only 5 min (Fig. 1b). The latter shows that the analytical method is highly selective for the analytes of interest.

**Fig. 2:**
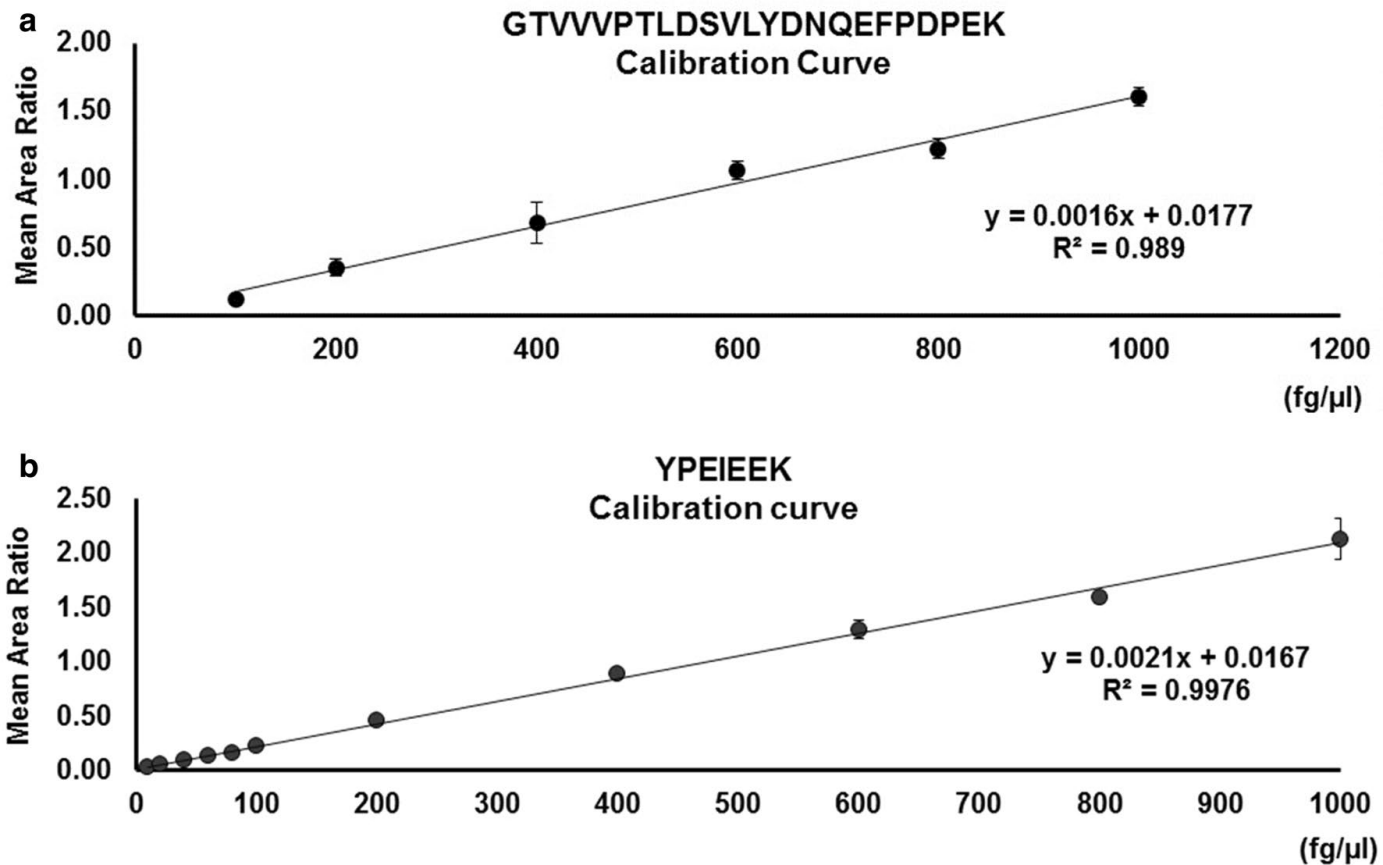
Calibration curves for CYP2E1 specific tryptic peptides YPEIEEK (P1) and GTVVVPTLDSVLYDNQEFPDPEK (P2). **a** YPEIEEK calibration curve was constructed from serial dilutions of synthetic peptides and spiked in pooled plasma from control subjects containing 50 fg µL^−1^ of deuterated YPEIEEK as internal standard. Each data point is mean of triplicates and error bars represent standard deviation (SD) from the mean. The calibration curve showed linearity (R^2^ = 0.997) between concentrations 0–1000 fg µL^−1^. **b** GTVVVPTLDSVLYDNQEFPDPEK calibration curve was constructed from serial dilutions of synthetic peptides and spiked in pooled plasma from control subjects containing 50 fg µL^−1^ of deuterated YPEIEEK as internal standard. Each data point is mean of triplicates and error bars represents standard deviation (SD) from the mean. The calibration curve showed linearity (R^2^ = 0.989) between concentrations 0–1000 fg µL^−1^

Partial validation of the optimized chromatographic method showed P1 is a reliable representative peptide of CYP2E1 (Table 1) for the intended purpose of this analytical method. P2 showed unsatisfactory reproducibility and sensitivity in the ESI-LCMS/MS analysis. The normal reference limits for CYP2E1 protein in peripheral circulation has not been documented to date. Therefore, to visually estimate the linear range, six independent calibration curves for each representative peptide were set-up. Each calibration curve consisted of 20 individual concentrations ranging from 0 to 10,000 fg µL^−1^. The observed linear ranges for P1 and P2 were 5–100,000 fg µL^−1^ (Fig. 2a) and 100–1000 fg µL^−1^ (Fig. 2b), respectively.

**Table 1:** Summary of method validation data

Parameters	YPEIEEK (P1)			GTVVVPTLDSVLYDNQEFPDPEK (P2)			IS-P1	IS-P2
Linear range	5–100,000 fg/µl			100–12,500 fg/µl			NA	NA
Linearity	r^2^ = 0.991 ± 0.007			r^2^ = 0.998 ± 0.001			NA	NA
LLOQ	5 fg/µl			100 fg/µl			NA	NA
Specificity	CV = 4.0 ± 0.04 %			CV = 15.9 ± 0.42 %			CV = 2.42 ± 0.05 %	CV = 14.2 ± 0.57 %
Quality control:	L	M	H	L	M	H	NA	NA
Intra-day								
Accuracy, %	99.2	99.8	98.2	41.0	69.2	70.6	NA	NA
Precision, CV%	3.64	4.79	10.93	55.14	5.32	17.13	NA	NA
Inter-day								
Accuracy, %	91.75	105.08	90.09	70.8	82.6	84.14	NA	NA
Precision, CV%	9.76	4.56	0.77	42.54	12.62	20.13	NA	NA

The optimized analytical method for CYP2E1 quantification was validated by assessing the linear range, linearity, LLOQ, specificity, intra and inter-group accuracy and precision. *IS-P1* internal standard for P1; *IS-P2* internal standard for P2; *NA* not applicable. Coefficient of variation (CV, %) is presented as mean ± standard deviation. Coefficient of variation, means and standard deviations were calculated using the Sigma plot 11.2 software. Overall, the analytical method showed higher sensitivity and specificity for P1 (LLOQ = 5 fg µL^−1^; CV% = 4.0 ± 0.004) in comparison to P2 (LLOQ = 100 fg µL^−1^; CV% = 15.9 ± 0.42). Higher intra-day accuracy (L = 99.2 %; M = 99.8 %; H = 98.2 %) and precision (L: CV% = 3.64; M: CV% = 4.79; H: CV% = 10.93) were observed for quantification of P1 in comparison to P2 [(Accuracy) L = 41.0 %; M = 69.2 %; H = 70.6 % / (precision) L: CV% = 55.14; M: CV% = 5.32; H: CV% = 17.13]. Similarly, Inter-day accuracy for P1 (L = 91.75 %; M = 105.08%; H = 90.09 %) showed higher than 90 % accuracy for all levels of QC quantitated while P2 quantification showed slightly lower accuracy (L = 70.8 %; M = 82.6 %; H = 84.14 %) for all levels of QC in comparison to P1. Inter-day precision for quantification of P1 at all 3 QC levels showed CV% < 10 showing that the quantification method was highly precise for P1. P2 showed poor precision (L: CV% = 42.54 %; M: CV% = 12.62 and H: CV% = 20.13 %) for L and H QC levels. Overall, P1 showed higher reproducibility and reliability as representative peptide for CYP2E1 in the optimized targeted proteomic approach

**Fig. 3:**
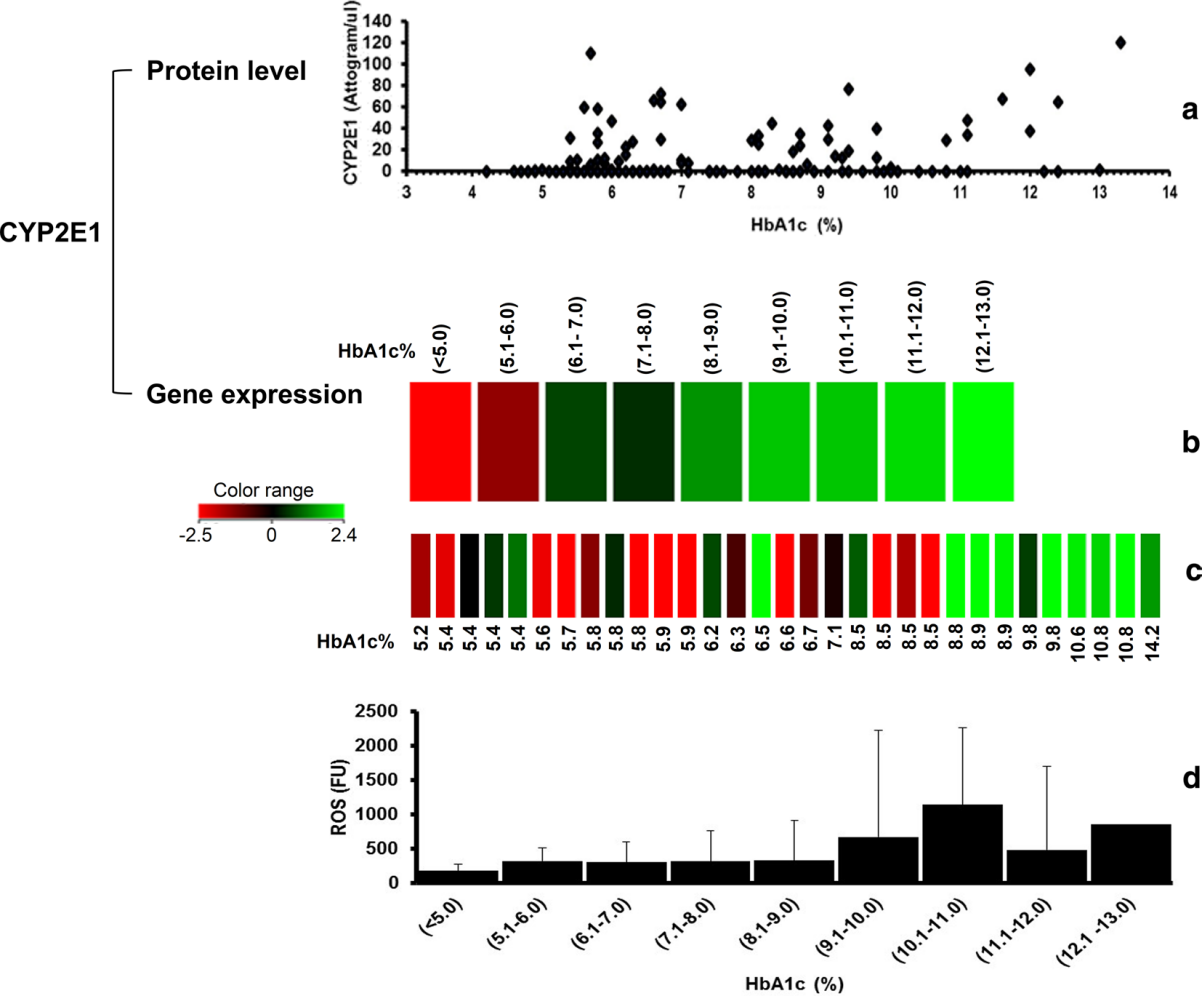
Correlation of CYP2E1 protein levels, CYP2E1 gene expression and ROS, with glycemic control. **a** A *scatter plot* generated using the SigmaPlot 11.2 software, presenting measurable amounts of CYP2E1 protein in individual subjects (n = 166). Data from this *scatter plot* shows measurable amounts of CYP2E1 were seen even when HbA1c was 4.2 %. **b** CYP2E1 gene expression presented as a heat map generated using the GeneSpring 12.6 software. The heat map shows averaged CYP2E1 gene expression of study subjects who were grouped according to their HbA1c percentage. The averaged CYP2E1 gene expression for the groups of subjects who had HbA1c [(< 5.0 %); n = 8] and [(5.1–6.0); n = 20] showed down regulation and groups for subjects with HbA1c percentage [(6.1–7.0); n = 19], [(7.1–8.0); n = 11], [(8.1–9.0); n = 20], [(9.1–10.0); n = 17],[(10.1–11.0); n = 9], [(11.1–12.0); n = 6], [(12.1–13.0); n = 6] showed up-regulation. The gradual change in hues of *green* from *dark green* for group (6.1–7.0) to the *lightest green* in group (12.1–13.0) shows the increments in up regulation intensities of CYP2E1 gene as the HbA1c percentage increases. **c** CYP2E1 gene expression presented as a heat map generated using the GeneSpring 12.6 software. The heat map shows CYP2E1 gene expression for individual subjects with various HbA1c percentages. The heat map shows that CYP2E1 gene is up regulated even when HbA1c was 5.4 %, which is well within the normal reference limit for HbA1c in clinical practice. **d** A *bar chart* representing average ROS levels in the groups of subjects who had HbA1c [(< 5.0 %); n = 8],[(5.1–6.0); n = 20], [(6.1–7.0); n = 19], [(7.1–8.0); n = 11], [(8.1–9.0); n = 20], [(9.1–10.0); n = 17], [(10.1–11.0); n = 9], [(11.1–12.0); n = 6] and [(12.1–13.0); n = 6]. *Error bars* represent standard deviations (SD) within each group. SD was calculated using the Sigma Plot 11.2 software. Overall, the increment in ROS levels showed increment trends with increasing HbA1c percentage similar to CYP2E1 gene expression and protein amounts. This observation strongly supports the notion that the increase in ROS production during hyperglycemia induces up-regulation of CYP2E1 gene expression in peripheral lymphocytes and subsequently increases in CYP2E1 protein

CYP2E1 is a non-abundant protein in human peripheral blood. Current study showed that CYP2E1 was not quantifiable in individuals with normal blood sugar levels (euglycemia) but was quantifiable in some of the pre-diabetic and most of diabetic subjects at varying amounts (Fig. 3a). The mean values for Pre-D and D groups were significantly different from the control group (C) (*p* < 0.001). No significant differences were seen between the means of Pre-D and D (*p* = 0.870). CYP2E1 gene expression correspondingly showed up regulation when HbA1c was still within normal reference limits (Fig. 3b, c).

HbA1c is the gold standard point-of-care test for evaluating blood glucose levels and it is globally used by clinicians for diagnosis of diabetes as well as for evaluating glycemic control alongside fasting blood glucose. When blood glucose exceeds homeostasis level it naturally binds to haemoglobin to form glycosylated hemoglobin (HbA1c). HbA1c values indicate average blood glucose levels for the past 2–3 months from the day HbA1c is tested on the patient [18]. The HbA1c test does not reflect on patients glycemic status beyond 3 months from the time of current test, unless HbA1c was tested once every 3 months repetitively. HbA1c levels continue to rise when blood sugar increases and decreases when patients consume oral antihyperglicemic drugs or when they undergo lifestyle modification as often prescribed by physicians. With regard to the latter, HbA1c does not represent cellular glucose metabolism processes during the hyperglycemia milieu which precedes the increase in blood glucose levels. Therefore, HbA1c values do not predict risks of diabetic complications.

Several studies commonly explained that within the hyperglycemic milieu ROS is grossly generated and promotes elevation of CYP2E1 protein levels [8–10]. CYP2E1 itself has oxidative properties [19] and along with glucose auto-oxidation is capable of generating more ROS which leads on to a positive turnover of ROS and CYP2E1; hence, enhancing a destructive environment at cellular level.

Measurement of ROS per se may not be suitable as an assessment of hyperglycemic related damage risk or severity of hyperglycemia. This is because the increase in ROS at any time may be transient due to the interplay of environmental factors, diet and various oxidants generated during normal intracellular metabolism. All these factors contribute to large inter-individual variations within each study group (Fig. 3d). The normal reference limit for ROS in human is ≤ 310 FORT units (FORT = free oxygen radical test) [20]. Moreover, in the human body an efficient natural antioxidant defence mechanism exists which aims to maintain physiological levels of ROS [21]. An imbalance of ROS homeostasis takes place during the onset of diabetes bias towards ROS generation at cellular level [17]. The latter processes create a toxic-driven catastrophic environment which hypothetically triggers the up-regulation of CYP2E1 to play its physiological role to detoxify the environment. Consistent with diabetes being a systemic disease it is possible that this initial up-regulation of CYP2E1 gene occurs in peripheral lymphocytes and the CYP2E1 protein becomes quantifiable in peripheral lymphocytes. CYP2E1 itself has oxidative properties [1] therefore, sustained increment in CYP2E1 will not be a favourable condition and together with auto-oxidation of glucose the cellular environment becomes destroyed and can contribute to multiple forms of pathogenesis.

Therefore, an undiagnosed diabetic patient who has abnormal HbA1c level at the first visit to a clinician may already be at risk for diabetic complications. According to clinical guidelines [22], HbA1C ≥ 6.5 % is equivalent to normal blood glucose level, but our data showed that CYP2E1 protein was quantifiable in some individuals even when HbA1C was < 6.5 %. On average, CYP2E1 protein (Fig. 3a) as well as CYP2E1 gene expression (Fig. 3b, c) were higher in diabetic individuals who had higher glycemic levels (HbA1c). Findings from this study clearly suggest that the appearance of CYP2E1 protein in peripheral lymphocytes precedes glycosylation of haemoglobin. Data from this study also suggest that the conventional clinical criteria [22] for diagnosis of diabetes based on HbA1c and fasting blood glucose alone may prevent early recognition of the risks of onset of diabetes and possibly its complications.

Quantification of CYP2E1 in diabetic patients gives a prediction of cumulative of oxidative damage and toxicity at cellular level due to hyperglycemia at the time the patient is tested for this marker. Since CYP2E1 was not detected in control subjects, the lowest quantifiable amounts during hyperglycemia should be regarded as a risk factor for diabetic complications because CYP2E1 is catastrophic by nature and is capable of exacerbating cellular damage and can be potentiated by other extrinsic and genetic factors.

With complete validation of our optimized targeted proteomic approach method, quantification of CYP2E1 in peripheral blood will be useful as an additional tool alongside current clinical indices for predicting risk of developing diabetes in patients with impaired glucose tolerance or impaired fasting glucose as well as for predicting risks for diabetic complications.

Our findings complement and strengthen previous discussions [17] on the role of ROS in diabetes progression. Data from this study clearly provide evidence that the amounts of ROS correlates incrementally with the severity of hyperglycemia indicated by increasing HbA1c levels (Fig. 3d).

## Conclusions

Consistent with the aims of our study, we evidently showed that the optimized targeted proteomic approach for quantifying CYP2E1 in peripheral blood is suitable for clinical application. “YPEIEEK” is unique to CYP2E1 and does not carry any polymorphic regions. “YPEIEEK” showed high inter-day and intra-day accuracy and precision. Therefore, “YPEIEEK” is a suitable and reliable probe for quantification of CYP2E1 in the general population.

The increment of CYP2E1 protein was consistent with increment of HbA1c values (gold standard marker for glycemic control) in peripheral blood, but the former precedes abnormal HbA1c values. This observation evidently suggests that CYP2E1 protein levels in peripheral blood is a potential candidate biomarker for risk of developing diabetes and probably its complications in prolonged hyperglycemia.

Data from this study showed in a cohesive manner, the amounts of circulating CYP2E1 protein amounts and gene expression in peripheral blood increases in parallel with ROS. This finding compliments and strengthens the notion that ROS is increasingly being generated during hyperglycemia and subsequently influences the up-regulation of CYP2E1 gene and protein expression in peripheral lymphocytes.

## Methods

### Materials

Reagents were obtained from Sigma Chemicals Co. (St. Louis, MO) and Agilent Technologies (M) Pte. Ltd. unless otherwise stated. Recombinant human CYP2E1 expressed in *Escherichia coli* was from Euro Science (M) Pte. Ltd. Synthetic peptides “YPEIEEK (P1)” and “GTVVVPTLYDNQEFPDPEK (P2)” were ordered from First BASE Laboratories (M) Pte. Ltd. LCMS analytical column was purchase from Agilent Technologies (M) Pte. Ltd.

### Research design and study subjects

This study was a cross sectional study. Study population (n = 166) consisted of three groups: normal healthy individuals (Control; n = 45), pre-diabetes (Pre-D; n = 25), and diabetes (D; n = 96). Our inclusion criteria were adult males and females aged 30–60 years and non-smokers. Our exclusion criteria were pregnant women and individuals who just recovered from any form of illness or surgery. Study subjects were grouped based on fasting plasma glucose levels (FPG), HbA1c and oral glucose tolerance test (OGTT) with reference to the American Diabetes Association (ADA) criteria for diagnosis of diabetes mellitus [23]. Control subjects (C) were individuals with fasting plasma glucose FPG < 5.6 mmol L^−1^ and/or HbA1c < 6.5 % on the recruitment day. The OGTT test reveals individuals with impaired fasting glucose (IFG) and impaired glucose tolerance (IGT). IFG is when plasma glucose level after an overnight fast (8 h) is ≥ 7.0 mmol L^−1^. IGT is when an individual has plasma glucose level ≥ 11.0 mmol L^−1^ 2 h after consuming 75 g glucose. Individuals with IFG or IGT were assigned to the pre-diabetes group (Pre-D). Individuals who had 2 h post prandial glucose ≥ 7.8 < 11.0 mmol L^−1^ or FPG ≥ 6.7 < 7.0 mmol L^−1^ and/or HbA1c ≥ 5.7 < 6.5 % were assigned to the Pre-D group [23]. The diabetes group (D) consisted of individuals who had fasting plasma glucose (FPG) ≥ 7.0 mmol L^−1^ and/or HbA1c ≥ 6.5 %, individuals with both IFG and IGT and individuals who are already on antihypertensive medication. All subjects were instructed not to consume any form of alcoholic beverages or food for 7 days before phlebotomy. Subjects who had any major health conditions within 5 months from the date of phlebotomy were excluded from participation. Characteristics of our study population are summarized in Table 2. Informed written consent was obtained from all subjects following human research ethics approval from the Ministry of Health Malaysia, Medical Research and Ethics Committee (NMRR: 12-501-11926). This study conforms to the Declaration of Helsinki.

**Table 2:** Characteristics of study population

Variables	Study groupings		
	C (n = 45)	Pre-D (n = 25)	D (n = 96)
Gender			
M	12	12	47
F	33	13	49
Age (years)	42 ± 13	50 ± 11	54 ± 9
HbA1c (%)	4.4 ± 0.36	6.0 ± 0.39	8.7 ± 2.0
FBS (mmol L^−1^)	4.9 ± 0.48	6.0 ± 1.16	10.0 ± 4.68
CYP2E1 (ag µL^−1^)			
	0 ± 0	7.66 ± 16.39	10.0 ± 20.79
* p*	C vs Pre-D: *p* < 0.001 C vs D: *p* < 0.001	Pre-D vs D: *p* = 0.905	

Data are presented as mean ± SD

*C* control, *Pre-D* pre-diabetes, *D* diabetes, *m* male, *f* female, *FBS* fasting blood sugar

### Sample collection

All subjects were instructed to fast overnight and phlebotomy was performed the following morning on each subject at our clinical research center (Monash University Medical Precinct). Approximately 8 µL of venous blood was collected into tubes containing EDTA anticoagulant. All phlebotomy sessions were between 8 a.m. till 11 a.m.

### Selection of CYP2E1- specific tryptic peptides

CYP2E1 unique peptides were selected in silico [24, 25] using the Agilent Spectrum Mill peptide selector (Revision: 3.3.084). Tryptic peptides which are portions of the membrane spanning region of CYP2E1 and those containing natural variant amino acid sequences were identified and excluded using the UniProt software http://www.uniprot.org/uniprot/PO5181. Subsequently, “YEPIEEK” and “GTVVVPTLDSVLYDNQEFPDPEK” were selected as suitable unique analytical target peptides to quantify CYP2E1.

### Isolation of lymphocytes from venous blood

Peripheral blood lymphocytes were isolated using NycoPrep media (Axis-Shield, Oslo, Norway) according to an established method [26]. 8 ml whole blood from each subject was diluted with equal volume of 0.9 % NaCl in a 50 mL Falcon tube. 16 µL of diluted blood was then carefully layered on 8 ml NycoPrep media in another 50 µL Falcon tube. Falcon tubes were then capped to prevent the formation of aerosols. All tubes were then centrifuged at 800×*g* for 20 min at room temperature in a swing out rotor (Beckman, Allegra X-12, USA). After centrifugation, the lymphocytes form a distinct band at the interface between the sample layer and the NycoPrep media. A pasture pipette was used to transfer the lymphocytes suspension into 15 µL glass centrifuge tubes without removing the upper layer. Equal volume of phosphate buffered saline (PBS) was added into centrifuge tubes containing lymphocytes suspension, mixed by gently inverting the tubes until the lymphocytes were homogenously diluted. Next, all tubes were centrifuged at 400×*g* for 10 min at room temperature. Supernatants were discarded and lymphocytes were resuspended in PBS and centrifuged again at 400×*g* for 10 min at room temperature. Supernatants were discarded and 1 µL 0.1 M Tris–HCl pH 7.4 was added into each tube, mixed gently to dislodge the lymphocytes and bring them to a homogenous suspension. All lymphocyte suspensions were then transferred into pre-labeled 1.5 µL microfuge tubes and immediately stored at −80 °C until used for microsome preparation.

### Preparation of lymphocyte microsomes

Microsomes were prepared according to established method [27] with slight modifications. Isolated lymphocytes stored at −80 °C were thawed gradually at room temperature, mixed gently by tapping at the bottom of the microfuge tubes and placed on ice. Lymphocytes were disrupted by sonication for 3 × 60 s on ice, followed by centrifugation at 10,000×*g* for 30 min at 4 °C (Beckman Coulter, Optima L-100 XP Ultracentrifuge). The supernatants were transferred into new microcentrifuge tubes, and centrifuged again at 100,000×*g* for 90 min at 4 °C. From this step onwards, all supernatants were discarded and the pelleted microsomes were resuspended in 100 mM sodium pyrophosphate buffer, pH 7.4, to inhibit the NADPH destructing pyrophosphatase activity of the microsome preparation. This was followed by another centrifugation at 100,000×*g* for 60 min at 4 °C. The washed microsomes were resuspended in 50 mM potassium phosphate buffer, pH 7.4, containing 0.1 mM EDTA, 0.1 mM dithiothreitol (DTT) and 20 % glycerol. Microsome suspensions were transferred into 1.5 µL microcentrifuge tubes and stored immediately at −80 °C until used for subsequent protocol.

### Isolation of soluble CYP2E1

CYP2E1 is a membrane bound protein. To facilitate the analysis and quantification this protein is enriched and solubilized in order to maximize cleavage by trypsin during the in-solution digestion procedure. CYP2E1 tryptic peptides were extracted similar to the procedure described by Liao et al. [28] with slight modifications to gain maximum recovery. Briefly, lymphocyte microsomes were sonicated (Sonicator 3000, Misonix Inc., Farmingdale, NY) followed by centrifugation at 153,000×*g* for 30 min. Lymphocyte lysate protein concentration was determined using Pierce bicinchoninic acid (BCA) protein assay kit (Thermo Scientific, IL, USA). Membrane bound proteins were solubilized by placing the resulting total membrane pellets in 0.2 % sodium cholate in 25 mM NH_4_HCO_3_ and vortexed continuously at 700 rpm for 60 min at room temperature. Subsequently, the dissolved membrane proteins were heated at 90 °C for 5 min to denature the solubilized membrane proteins to make them amenable to cleavage by trypsin. All samples were allowed to cool at room temperature, then treated with trypsin (Sigma-Aldrich, catalog number: T0303) for 15 h at 37 °C. The substrate to trypsin ratio used was 50/1 (w/w). After hydrolysis, 50 fg µL^−1^ internal standard peptides (deuterated “GTVVVPTLDSVLYDNQEFPDPEK” and “YPEIEEK”) were added to each sample and centrifuged at 153,000×*g* for 30 min. Supernatants for each sample were transferred to clean tubes and equal volumes of acetonitrile were added. All samples were dried using a Vacufuge (Eppendorf AG, Hamburg, Germany).

### Analysis and quantitation by MRM using UHPLC-ESI- LC–MS/MS

Analytical parameters for quantitation of target peptides were optimized on the Agilent 1100 UHPLC system (Agilent, Santa Clara, USA) which was connected to an ESI triple quadrupole mass spectrometer (Agilent 6410). The targeted unique peptides were separated on ZORBAX Eclipse Plus C8 Rapid Resolution column (Agilent, RRHD; 2.1 × 50 mm; 1.8 µm particles) and eluted using a non-linear mobile phase schedule. The mobile phase consisted of 0.1 % formic acid in water (solvent A) and 0.1 % formic acid in 90 % acetonitrile/water (solvent B).

### Analytical method validation

#### Linearity and range

Synthetic peptides (YPEIEEK, and GTVVVPTLDSVLYDNQEFPDPEK) were serially diluted to obtain final concentrations of 1000, 800, 600, 400, 200, 100, 80, 60, 40, 20, 10, 5 and 0 fg µL^−1^ in pooled plasma from normal volunteers containing 50 fg µL^−1^ of each internal standard. Triplicates of each calibrator was injected into the LCMS/MS system to generate standard curves (n = 6).

#### Specificity

Synthetic peptides and internal standard peptides were prepared as a mixture containing 30 fg µL^−1^ of each peptide in pooled plasma from normal volunteers. 1 µL of peptide mixture was injected into the LCMS/MS system. The injections were repeated 10 times in the same day. Specificity was evaluated by comparing the repeatability of retention times of each peptide [29, 30].

#### Accuracy

Three levels of quality control (QC) standards for each peptide (10, 30 and 60 fg µL^−1^) containing 50 fg µL^−1^ of each internal standard were prepared in pooled plasma from normal volunteers. Triplicates of each QC standards were injected and data were collected for 6 runs. The observed values for each QC were then predicted from a 7 points calibration curve (500, 250, 125, 50, 25, 5, 0 fg µL^−1^). Percentage of error (bias) was calculated as: $Error\ \left( \% \right) = \frac{Expected\; value - Observed\; value}{Expected\; value} \times 100$. Percentage of accuracy was calculated by subtracting error (%) value from 100 %.

#### Precision

Precision was assessed by comparing the peak area ratios from 6 runs of QC standards (3 levels) for each peptide. Peak area ratio is calculated by dividing the area under the curve (AUC) for analyte peptide by AUC for internal standard peptide [29, 30]. Preparations of QC standards were similar to the QCs prepared for accuracy evaluation. Mean (n = 6) percentage coefficient of variation (CV) for each QC should be ≤15 % [29, 30].

#### Inter-day precision

The procedure for precision and accuracy assessment was repeated for 6 days and the percentage of CV for 6 days was calculated.

#### Intra-day precision

The procedure for precision and accuracy assessment was repeated for 6 runs in a single day and the percentage of CV was calculated for observation.

### Measurement of reactive oxygen species (ROS)

ROS was measured using a free oxygen radical monitor (FORM-OX; Callegari, Italy).

### Measurement of HbA1c

Glycosylated hemoglobin (HbA1c) was measured using Afinion AS100 analyzer (Axis-Shield, USA) according to manufacturer’s protocol. This instrument conforms to the National Glycohemoglobin Standardization Program (NGSP) and diabetes control and complications trial (DCCT) standards.

### CYP2E1 gene expression

Total RNA was extracted from each sample using RNeasyPlus Micro Kit (Qiagen, Germany) according to manufacturer’s protocol. Purity (*A*_260_/*A*_280_), RNA integrity number (RIN) and concentration of extracted total RNA were assessed using Agilent 2100 Bioanalyzer with RNA 6000 Nano Reagents (Agilent Technologies, Santa Clara, USA). Only samples with RIN 7.0 or higher were selected for the gene expression experiment. 100 ng of total RNA of each sample was processed and hybridized to SurePrint G3 Human Gene Expression Microarray platform (G4858A; Agilent Technologies, Santa Clara, USA) according to manufacturer’s protocol. Microarray slides were scanned using Agilent G4900DA Sure Scan Microarray Scanner (Agilent Technologies, Santa Clara, USA). After scanning the image data files were imported into the GeneSpring GX gene expression software, Version 12.6 (Agilent Technologies, USA) for detailed analysis.

### Statistical analysis

Significant difference in CYP2E1 protein levels between the studied groups (C, PD, D) were calculated using SigmaPlot 11.2 software. Since our study samples were not drawn from normally distributed populations with the same variances, we used the Mann–Whitney rank sum test. Statistical analysis was performed in a pair-wise manner (C versus PD; C versus D and PD versus D). Differences between study groups were considered significant at *p* < 0.05. Intra-group variations for variables in subjects and study parameters were described as standard deviations (SD). SDs were also calculated using the SigmaPlot statistical software.

## References

[1] Danielson PB (2002). The cytochrome P450 superfamily: biochemistry, evolution and drug metabolism in humans. Curr Drug Metabolism..

[2] Villeneuve JP (2004). Pichette. Cytochrome P450 and liver disease. Curr Drug Metab..

[3] Molina-Ortiz D, Camacho-Carranza R, Gonzalez-Zamora JF, Shalkow-Kalincovstein J, Cardenas-Cardos R, Nosti-Palacios R, Vences-Mejia A (2014). Differential expression of cytochrome P450 enzymes in normal and tumor tissues from childhood rhabdomyosarcoma. PLOS ONE..

[4] Rodriguez-Antona C, Ingelman-Sundberg M (2006). Cytochrome P450 pharmacogenetics and cancer. Oncogene.

[5] Oyama T, Kagawa N, Kunugita N, Ogawa M, Yamaquchi T, Suzuki R, Kinaga T, Yashima Y, Ozaki S, Isse T, Kim YD, Kim H, Kawamoto T (2004). Expression of cytochrome P450 in tumor tissues and its association with cancer development. Front Biosci..

[6] Wang Z, Hall SD, Maya JF, Li L, Asqhar A, Gorski JC (2003). Diabetes increases the in vivo activity of cytochrome P450 2E1 in humans. Br J Clin Pharmacology..

[7] Hannon-Fletcher MP, O’Kane MJ, Moles KW, Barnett YA (2001). Lymphocyte cytochrome P450-CYP2E1 expression in human IDDM subjects. Food and Chemical Toxicology..

[8] Haufroid V, Ligocka D, Buysschaert M, Horsmans Y, Dominique L (2003). Cytochrome P4502E1 (CYP2E1) expression in peripheral blood lymphocytes: evaluation in hepatitis C and diabetes. Eur J Clin Pharmacol..

[9] Yu SY, Chung CH, Kim EJ, Kim SH, Lee I, Kim SG, Lee MG (2002). Effects of acute renal failure induced by uranyl nitrate on the pharmacokinetics of intravenous theophylline in rats: the role of CYP2E1 induction in 1, 3-dimethyluric acid formation. J Pharm Pharmacol..

[10] Christina GY, Zaini A, Othman I (2013). CYP2E1 level in peripheral lymphocytes correlates with early pathogenesis of diabetic nephropathy. Biomed Res.

[11] Murray M (2006). Altered CYP expression and function in response to dietary factors: potential roles in disease pathogenesis. Curr Drug Metab.

[12] Galeva N, Altermann M (2002). Comparison of one-dimensional and two-dimentional gel electrophoresis as a separation
tool for proteomic analysis of rat liver microsomes: cytochromes P450 and other membrane proteins. Proteomics..

[13] Surapaneni KM, Priya VV, Mallika J (2014). Pioglitazone, quercetin and hydroxyl citric acid effect on cytochrome P4502E1 (CYP2E1) enzyme levels in experimentally induced non alcoholic steatohepatitis (NASH). Eur Rev Med Pharmaco Sci..

[14] Martinez-Gil N, Flores-Bellver M, Atienzar-Aroca S, Lopez-Malo D, Urdaneta AC, Sancho-Pelluz J, Peris-Martinez C, Bonet-Ponce L, Romero FJ, Barcia JM (2015). CYP2E1 in the human retinal pigment epithelium: expression, activity and induction by ethanol. Investigative Ophthalnology & visual science..

[15] Seibert C, Davidson BR, Fuller BJ, Patterson LH, Griffiths WJ (2009). Wang Yuqin. Multiple-approaches to the identification and quantification of cytochromes P450 in human liver tissue by mass spectrometry. J Proteome Res.

[16] Sato Y, Miyashita A, Iwatsubo T, Usui T (2012). Simultaneous absolute protein quantitation of carboxylesterases 1 and 2 in human liver tissue fractions using Liquid Chromatography-Tandem Mass Spectrometry. Drug Metabo Dispos.

[17] Kangralkal VA, Shivraj DP, Bandivadekar RM (2010). Oxidative stress and diabetes, a Review. Int J Pharm Appl.

[18] American Diabetes Association (2010). Diagnosis and classification of diabetes mellitus. Diabetes Care..

[19] Kessova I, Cederbaum I (2003). CYP2E1: biochemistry, toxicology, regulation and function in ethanol-induced liver injury. Curr Mol Med..

[20] Palmieri B, Sblendorio V (2007). Oxidative stress tests: overview on reliability and use. Eur Rev Med Pharm Sci.

[21] Johansen JS, Harris AK, Rychly DJ, Ergul A (2005). Oxidative stress and the use of antioxidants in diabetes: linking basic science to clinical
practice. Cardiovasc Diabetol..

[22] American Diabetes Association (2012). Standards of medical care in diabetes 2012. Diabetes Care..

[23] American Diabetes Association (2010). Diagnosis and classification of diabetes mellitus. Diabetes Care.

[24] Kawakami H, Ohtsuki S, Kamiie J, Suzuki T, Abe T, Terasaki T (2011). Simultaneous absolute quantification of 11 cytochrome P450 isoforms in human liver
microsomes by liquid chromatography tandem mass Spectrometry with *in-silico*
target peptide selection. J Pharm Sci.

[25] Kamiie J, Ohtsuki S, Iwase R, Ohmine K, Katsukura Y, Yanai K, Sekine Y, Uchida Y, Ito S, Terasaki T (2008). Quantitative atlas of a highly sensitive simultaneous LC/MS/MS method combined with novel in-silico peptide selection criteria. Pharm Res.

[26] Boyum A, Brincker F, Martinsen T, LØvhaug D (2002). Separation of human lymphocytes of human lymphocytes from citrated blood by density gradient (NycoPrep) centrifugation: monocyte depletion depending upon activation of membrane potassium channels. Scand J Immunol..

[27] Raucy JL, Schultz ED, Wester MR, Arora S, Johnston DE, Omdahl JL, Carpenter SP (1996). Human lymphocyte cytochrome P450 2E1, a putative marker for alchohol-mediated changes in hepatic chlorzoxazone activity. Drug Metab Dispos..

[28] Liao WL, Heo GY, Dodder ND, Pikuleva IA, Turko IV (2010). Optimizing the conditions of a multiple reaction monitoring assay for membrane proteins: quantification of cytochrome P450 11A1 and adrenodoxin reductase in bovine adrenal cortex and retina. Anal Chem..

[29] Guidance for Industry: Bioanalytical method validation. U.S. Department of Health and Human Services, Food and Drug Administration; 2001. pp 1–25.

[30] Thompson M, Ellison SLR, Wood R (2002). Harmonized guidelines for single laboratory validation of methods of analysis. Pure Appl Chem..

